# The Occurrence of Acute Postoperative Confusion in Patients after Cardiac Surgery

**DOI:** 10.1100/tsw.2005.109

**Published:** 2005-10-28

**Authors:** Jürgen Osterbrink, John P. McDonough, Andre Ewers, Herbert Mayer

**Affiliations:** ^1^ School of Nursing, Klinikum Nürnberg, Heimerichstrasse 58, 90419 Nürnberg, Germany; ^2^ Institute for Nursing Sciences, Universität Witten/Herdecke, Germany; ^3^ School of Nursing, University of North Florida, Jacksonville, USA; ^4^ Anesthesiology Nursing, University of North Florida, Jacksonville, USA; ^5^ Universität Witten/Herdecke, Germany; ^6^ Institute for Nursing Sciences, Universität Witten/Herrdecke, Germany

**Keywords:** confusion, postoperative delirium, cardiac surgery

## Abstract

This study quantified the occurrence of acute confusion in cardiac surgery patients at three German hospitals. A total of 867 patients, 22–91 years old, were examined each nursing shift postoperatively for 5 days for the presence of acute confusion using a modified version of the Glasgow Coma Scale and Confusion Rating Scale. The night shifts and the third postoperative day showed the most frequent periods of occurrence. Confusional state was noted in patients ranging from 10.5% for patients aged <70, to 40.7% for patients >80 years of age. Those found at increased risk were patients of increasing age and coexisting disease. Targeted nursing interventions for patients at increased risk of acute confusion may decrease this complication.

## INTRODUCTION

The “acute confusional state” was discussed by Segatore et al.[1]. It has been difficult to arrive at a standardized definition of acute postoperative confusion[2,3,4]. The duration of acute confusion varies from individual to individual and can last from a few hours to up to several days. The cause of this clinical state has been, and remains, the subject of much speculation. Heller et al.[5] and Shaw et al.[6] suggested advanced age and a lower preoperative general state of health as factors predisposing patients toward the condition. Heller refers to a higher incidence of postcardiac surgery delirium patients with an acquired heart condition when compared with patients with a form of congenital heart disease (26% as opposed to 8%). This aspect is similarly highlighted by Hammon et al.[7]. Like Smith[8], they identify a connection between advanced age, a raised microembolism, arteriosclerosis, and “neurobehavioral deficits” after heart surgery intervention with the utilization of a cardiopulmonary bypass (CPBP).

As factors conducive to the onset of postoperative confusion, Morgan and Closs[9] point to the postoperative lack of sleep and the stress found in the intensive care unit, along with the “sensory monotony” associated with being a patient in an intensive care unit. Along with sleep deprivation, Freyhan et al.[10], Sveinsson[11], and Heller et al.[5] highlight the length of time during which the patient remains in the hospital and the "psychological climate" found on intensive care wards as other potential factors conducive to mental confusion. These units are often characterized a climate of both hectic and continuous crisis situations. Orr and Stahl referred to the staff on intensive care wards as paying little attention to noise levels and lighting conditions and to other factors, all of which may well play a role in interfering with postoperative recovery[12].

## BACKGROUND

As a result of all of these factors, the postoperative recovery phase may well be delayed. Freyhan et al.[10], as well as Zurbrügg et al.[13], surmise that such postoperative phase experiences must play a role in the onset of "postcardiotomy delirium" and they underline the required nature of the postoperative patients' dependence on the nursing and medical staff functioning as a team, working together to speed postoperative recovery and a return to optimal functioning as soon a possible. Among the patient group concerned, there are reports of a considerably increased level of morbidity as well as of an increased mortality rate and of consequently lengthened hospital stays[4,14,15]. With illness interfering with patients' ability to comply with instructions, they are often more susceptible to instabilities and infections of the sternum, to various forms of pneumonia, and to postoperative decubitus, and thus require a higher level of nursing care[17,18]. The level of nursing care required is further increased by inappropriate behavior such as attempting to get out of bed, pulling at various invasive lines, or disconnecting them[3,19,20]. The implications of the phenomenon of acute confusion are not confined to the areas of nursing and medical care. With the complications that accompany it and the rehabilitation measures required, this phenomenon also impacts on area health care economics[14]. There are indications that even after a stay in the hospital, on falling ill with acute confusion patients can succumb to difficult or irreversible cognitive disorders. Therefore, having information on the prevalence of the illness that depicts the problem as a whole, together with any calculations on the incidence that may shed light on the number of recent cases in this patient group, may be of importance and helpful across health care disciplines.

## METHODS

Prior to this study, no data could be identified through review of information available in PubMed, CINAHL, Psych-Lit, and reports of the Deutsche Kardiochirurgische Gesellschaft concerning either the prevalence or the incidence of postoperative confusion among heart surgery patients in Germany. The study was conducted in an effort to document both the incidence and the prevalence of acute confusion after elective heart surgery. The term "incidence" is used to quantify the number of episodes of confusion of the total study population, whereas "prevalence" is the quantification of subjects who displayed episodes of postoperative confusion. By using this procedure, data were obtained concerning not only the number of subjects who displayed this complication, but also the patterns related to its occurrence. The study was carried out in an attempt to answer the following questions:

- When did acute confusion condition begin after cardiac surgery?
- In what percentage of subjects does acute confusion occur after cardiac surgery?

The study was approved by the ethics committees at the appropriate institutions. The study was conducted as a prospective cohort design with measurement of both independent and dependent variables on the day of surgery and continuing through midnight on the fifth postoperative day. This period was chosen in view of previous studies, which suggest that acute confusion develops primarily in the first 6 days after surgery[2,20,21]. Subjects were recruited and informed consent obtained on the afternoon prior to the scheduled surgery. Once consent had been obtained, demographic data, health history, and medical records data were reviewed and recorded. Afterward, beginning with the afternoon of the operation day, the level of consciousness and disorientation were evaluated during each of three daily nursing shifts for the next 5 days.

Freyhan et al.[10], as well as Tune et al.[22] and Koolhoven et al.[23], defined confusion as the simultaneous presence of disorientation and disturbance of consciousness. Immer et al., however, in their study, asserted that if either a disturbance of consciousness or disorientation were present, the patient should be classified as confused. In this study, both symptoms were included[24]. Only when disturbance of consciousness and disorientation were both present was the subject classified as confused. Thus, the findings of both Rasin[19] and of Neelon[2] were taken into consideration. They envisaged that impairment of consciousness is only one variable and insisted that not only the level of consciousness, but also attentiveness and orientation, should be assessed in accordance with the degree of consciousness.

Levels of consciousness and of attentiveness were determined postoperatively using the modified form, as validated by Brodner, of the Glasgow Coma-Scale. This scale registers the size of a diminution of consciousness quantitatively by recording movement potentials such as the opening of the eyes and the best verbal and motor responses. These are converted into points of value[25]. The highest value that can be achieved, unimpaired consciousness, is 15. In this study, impairment of consciousness was determined to be present when the value fell below 13.

The Confusion Rating Scale was employed so that the four dimensions, namely “disorientation, temporal, local and spatial and inadequate behavior, as regards place and/or person,” “inadequate communicative ability”, where “place and/or person are concerned”, and lastly “illusion and hallucination” are taken into consideration[26]. William’s Scale was translated and in this study showed a correlation coefficient of $r$ = 0.88 following Spearman (confidence interval 0.81–0.93). The measure of internal consistency was, following Cohens Kappa, 0.81. The investigation of the variables for both instruments was carried out periodically by nursing staff on shift duty during the period of investigation on the various wards that supplied the patients. Since the study did not include any intervention on the patients who were taking part, no formal ethical issues arose regarding treatment or lack thereof. Declarations of consent were also obtained from the heads of each clinic.

The study was carried out over a 3-month period in three different heart surgical hospitals in northern and southern Germany. The chosen hospitals are affiliated teaching hospitals of the respective local universities. Every patient who met the entry requirements was recruited for the study. The patient suitability criteria were as follows:

- Patients had to be able to understand German and give consent to take part in the study.
- Patients were awaiting heart surgery, with or without the use of a heart-lung machine, and had already been fully briefed on this.
- Patients were required to be over 18 years of age and the length of their hospital stay had to cover, as a minimum, the entire period of the investigation.

A total of 876 patients met the entry criteria. Of these, 16 patients failed to complete the entire research period, either through failure to consent, death, or due to prolonged postoperative intubation.

In “Hospital A” (bed capacity of 643), 950 heart operations are performed each year, “Hospital B” (bed capacity 1300) performs 2800 heart operations, and “Hospital C” (bed capacity of 1500) carries out 2000 heart operations. A total of 45.4% of this study’s total participants were treated in “Hospital B”. The mean age of the sample group was 65.97 with outliers of 22 and 91. The lower and upper quartiles divided up as follows: 25–60.1 and 75–73.0. The median ranges from 60–73 and, thus, corresponds in age with the total sample group described in the Bruckenberger Heart Report as the group most relevant[27]. The patients enrolled in this study were 62% male and 38% female (see Fig. 1).

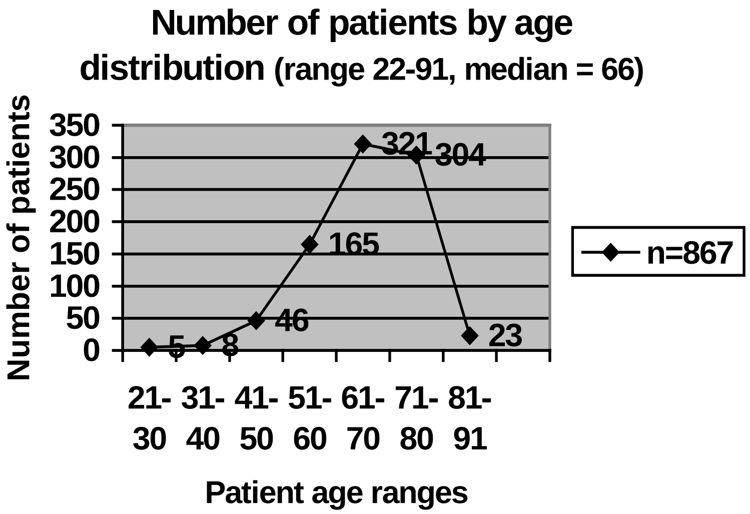

**FIGURE 1**.

## FINDINGS

In 97.1% of all the patients, the operations were performed using cardiopulmonary bypass; 76.6% (n = 664) of all patients received an aortic-coronary bypass. Valve replacement was performed on 18.5% (n = 160) patients. In 5.9% (n = 52), a combined intervention valve replacement and revascularization was carried out. A total of 10 patients, 1.1% of the patient group, underwent an emergency postoperative rethoracotomy. In 8.4% (74) of the cases, a venous revascularization was performed. Of the total, 188 patients (22.1%) were either type I or type II diabetics. Depending on the type of operation, 58.2% of the patients showed symptoms of confusion on the intensive care ward and 39.7% did so in intermediate care. In the general care unit, 2.1% patients displayed confusion.

In this study, incidence was calculated cumulatively. This basis for calculating includes all patients who were “at risk” at the beginning of the time period. If a patient showed symptoms of acute confusion in the investigation period and then, after a symptom-free interval, had confusion symptoms a second time, it was not recorded as a new appearance.

Of the sample group of 860 identified as being “at risk”, in the whole period, 152 (17.67%) of all patients developed symptoms of acute confusion during at least one nursing shift. The 95% confidence interval was between 15.18–20.39%. If incidence per day is described separately, it then becomes clear that the first appearance of the phenomenon in a total of 47 patients on the second postoperation day (6%) stands out when compared with a total of 42 patients (5%) on day 1 and with 14 patients (2.1%) on day 5.

Thus, acute confusion did not make the first appearance uniformly. In terms of absolute numbers, it was observed to have peaked on the second postoperation day. Patients who experienced acute confusion for the first time postoperatively had to be spotted above all in the first 72 h. At any rate, up to 1.63% of all 860 patients at risk were still showing first symptoms of the phenomenon as late as postoperation day 5. It was on the third postoperation night in particular that most confusion problems made their appearance, a percentage of 3.14, as depicted in Figs. 2 and 3.

This trend is confirmed in that in the period mentioned the incidence rate varied between 1.29 and 3.96%. The night shift was particularly significant. It displayed an incidence rate of 8.26%, in contrast with that of the day shift (4.77%) and that of the evening shift (3.84%) (see Fig. 4).

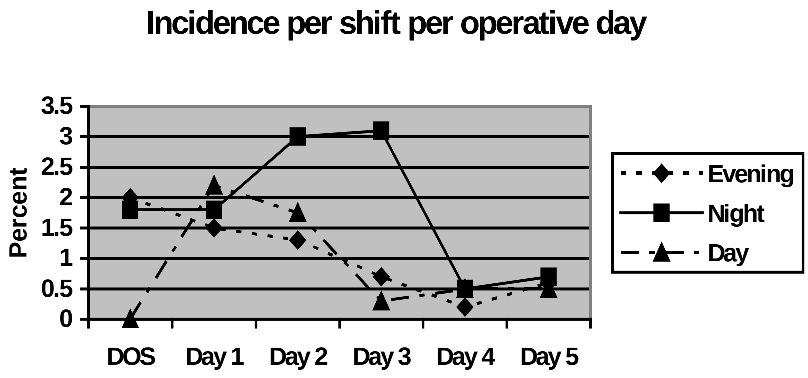

**FIGURE 2.**

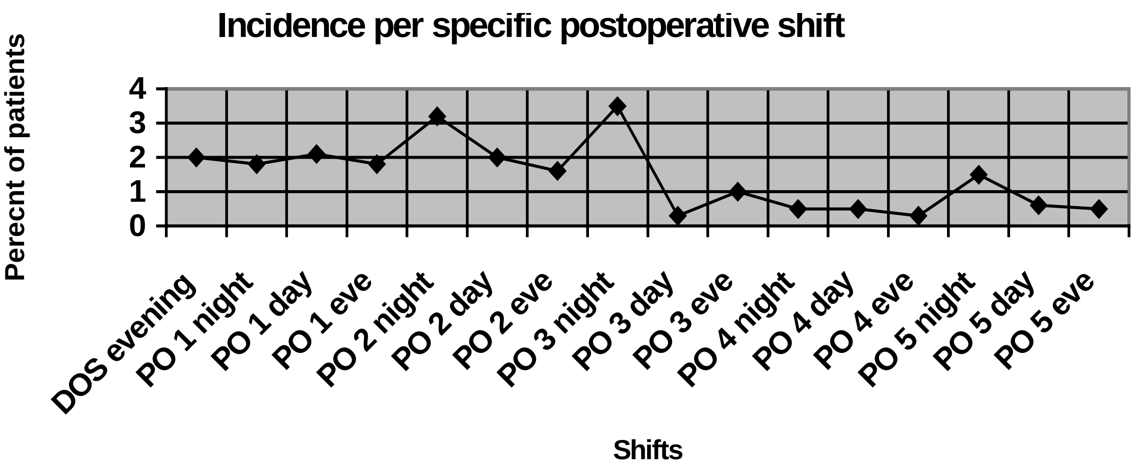

**FIGURE 3.**

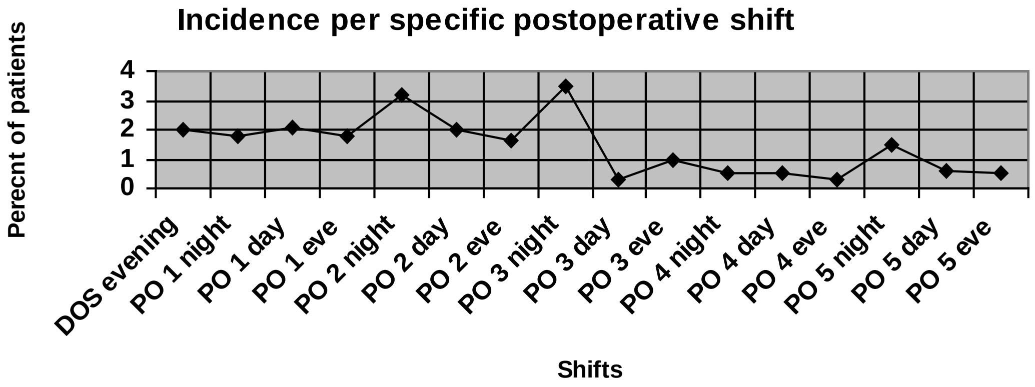

**FIGURE 4.**

The effect of age on the incidence of acute confusion has a variable significance of $p < 0.01$ between the individual age groups. This indicates that particular attention should be paid to the sample group of 75–79 year olds as well as to those in their eighties. However, even in the 70–74 age group, every fourth patient is affected. In this study, there were 17.7% male and 18.8% female, no differences in confusion were related to gender (see Figs. 5 and 6).

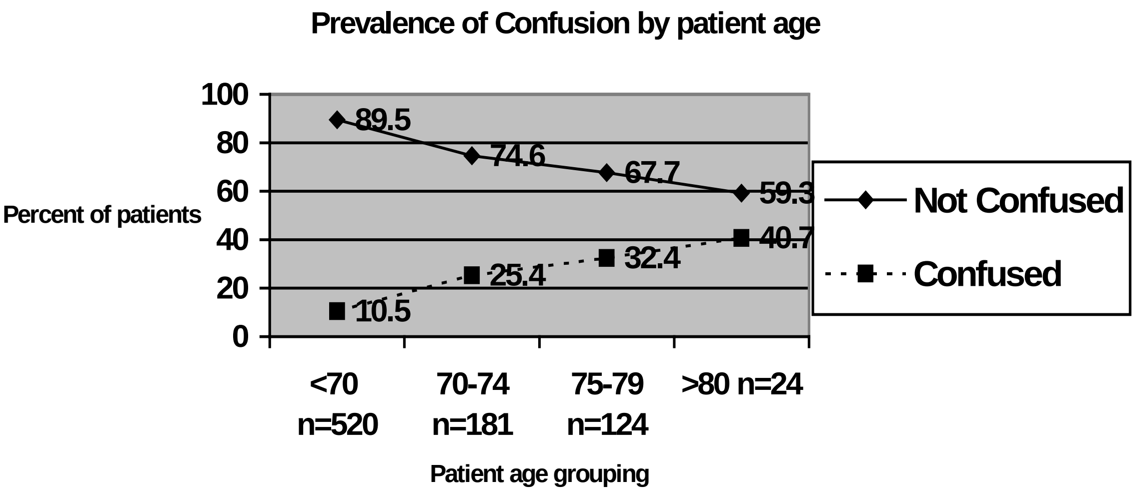

FIGURE 5.

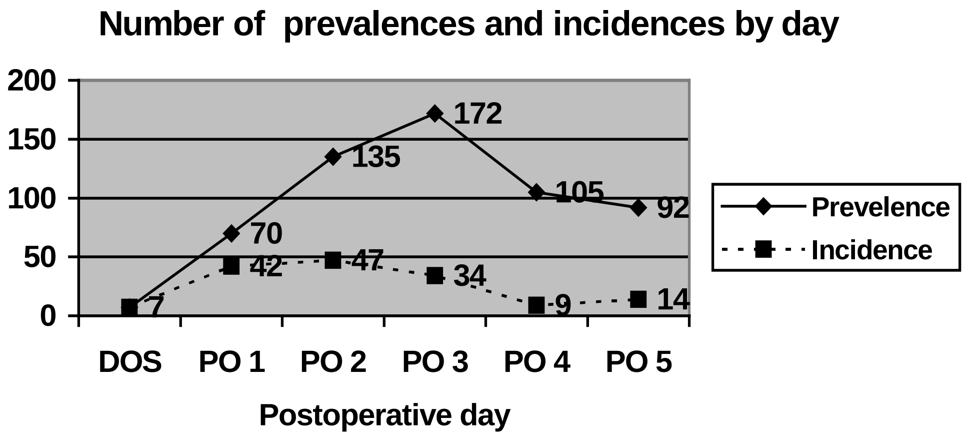

FIGURE 6.

In the period when measuring was taking place, the prevalence rate of postoperative confusion showed no stability. On the day of their operation, it was possible to find only seven patients who, on investigation, had postoperative confusion. By the end of the first postoperation day, the number of patients affected had increased tenfold, almost to double again by the end of postoperation day 2. The peak was reached on postoperation day 3 with 172 patients affected. On the fourth day of measuring, there was a significant fall (to 105 patients), as also on the fifth and final day, down to 92 patients.

In the diagram below, the course of the shifts shows that days 2–4 with occurrence rates of 6.2, 9.7, and 6.8%, respectively, require special attention. The third night shift is identified as the critical one. On postoperation day 1, a brisk rise in occurrence level is discernable. On day 2, the prevalence rate clearly increases likewise, to fall away after night 3 down to the level achieved at the end of the second late duty. A further, more gradual, fall is discernable. It becomes evident that when patients succumb to acute confusion, the symptoms remain for several days and that at the end of the measuring period (120 h), many patients still continue to suffer from confusion. The prevalence data show further that, all in all, the night shift (13.5%) compared to the early shift (11.6%) and the late shift (11.7%) points to a certain potential external stress load on both nursing and medical staff (see Fig. 7).

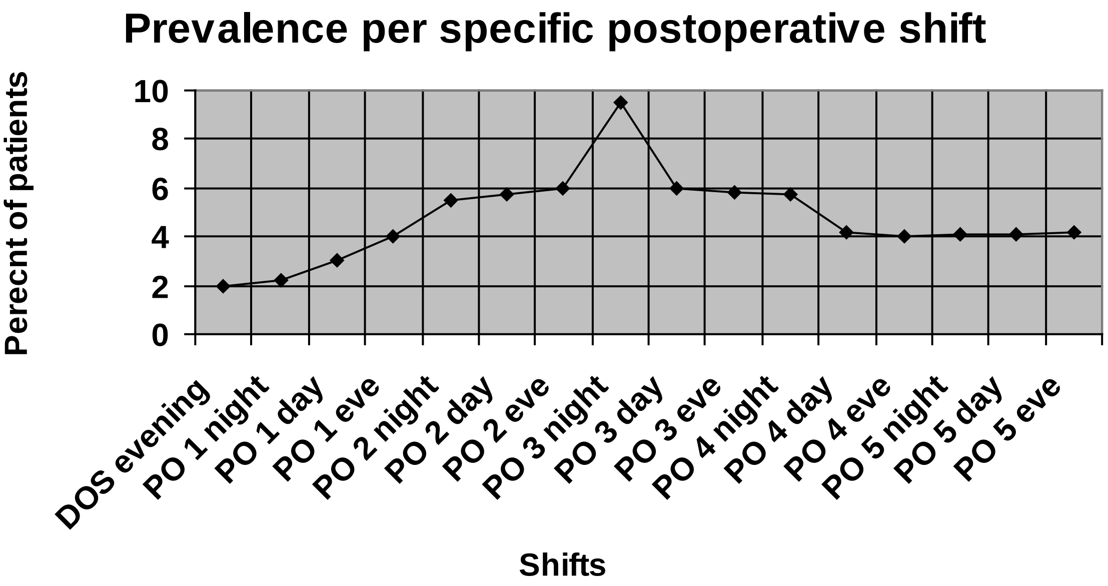

FIGURE 7.

## DISCUSSION

The total incidence of postoperative confusion fluctuates and is specified in the literature as being between 0 and 73%[4,28]. It is noteworthy that in orthopedic and heart surgery departments, conditions of acute postoperative confusion are considered as a matter of priority[29,30,31,32]. Immer et al. identified an incidence rate of from 3.2 to 5.1% in their studies of heart surgery patients (1299 and 438, respectively)[24].

In this study, the incidence fluctuated daily between 3.12 and 3.40%. One must bear in mind that in the research period as a whole, a total of 17.67% of all patients showed symptoms of confusion. It is worth noting that, although the criteria used in this study were modified and more stringently formulated than those in that of Immer, the incidence levels obtained are strictly comparable. In contrast to the accounts of others, in this study it was established that it is possible for acute confusion to emerge even 5 days after surgical intervention[4,21]. This actually happened to 14 (1.63%) of the total sample group. Confirmation was obtained through a process of regular, periodic recording. The confusion condition may make its first appearance at varying times. The first eight measurement periods, from the day of the operation until the night shift on day 3 are particularly worthy of note. Comparison of days 4 and 5 located an increase of 9 to 14 patients. This fact has implications for nursing staff no less than for medical staff in that “early warning systems”, through the purposeful collection of relevant data, can accurately register confusion conditions in a way similar to the checking of vital signs, thereby initiating specific interdisciplinary forms of therapy.

When the incidence rates are compared with the prevalence data, it becomes clear that prevalence peaks on postoperation day 3, then on postoperation day 5. At the final measurement point, 92 patients were still suffering from acute confusion. When these data are supplemented with data obtained daily from the shifts and a comparison is then made, it becomes evident that, granted the night shift’s outstanding 13.5% rate of occurrence, there is an overall burden imposed on patients and care teams alike. As a consequence, the recovery phase begins significantly later for postoperation patients, while that of the secondary illnesses already described is enhanced. These findings correspond to the delineations made by Neelon[2] and by Gallinat et al.[33], namely that the acute confusion condition can be especially prone to fluctuation and involves a significantly increased workload for the treatment team. It is for this reason that both of these clinical aspects, prevalence level and incidence portrayal, play a decisive role in enabling one to arrive at those kind of comprehensive actions that can safeguard quality of care. Acute postoperative confusion has consequences. Geary describes extended hospital stays of up to 13 days[34]. Several studies speak of an increased mortality rate among the patients affected and others discuss increased morbidity, demonstrable after a year-long investigation into its cause[1,15,33,34,35,36].

In this study, it is night that appears to have acquired a special significance. It was during the night shift that confusion conditions first made their appearance in 70 patients. Egerton and Kay[37] and Heller et al.[5] had already identified features conducive to the onset of confusion condition and, of these, Sveinsson[11] emphasizes the length of time spent in the intensive care unit as a factor most likely, in a number of cases, to promote mental confusion and postoperation delirium. Yet another feature is the suppression of sleep during the first night in the intensive care unit. Sveinsson maintains that many patients think they have not slept at all and others report having slept only a little. Patients who cannot see a clock believe they have been asleep for hours, although only 5 min have gone by. Such findings correspond with those of Williams et al. who labeled the phenomenon “sundowning”[38]. Amid other clinical phenomena, a similar course of development to that of postoperative pain can be discerned. As early as 1832, Sigmond described in the *Lancet* the phenomenon as “an illness that gnaws away at the whole anatomical system and that disrupts every bodily function while during darkness and the quiet solitude of the night destructive feelings turn to rebellion and hunt down the enfeebled spirit with redoubled energy.”

As a means of stress reduction, Sullivan et al. pointed to the management of the sleep cycle in so far as lighting conditions adapt to this cycle[39]. In the treatment of postoperative confusion, Depenbusch also thinks that a nightlight can play a significant role in reorientation at night[25]. She insists, however, that the main aim must be the continued maintenance of the sleep cycle.

In this study, it is the age of patients taking part that emerges as one of the determining factors. The 75–79 age group, along with that of the over eighties, were particularly affected by confusion, 32.2 and 40.7%, respectively. The findings of Kirsch et al., Ennabli and Pelletier, Okita et al., and Velanovich confirm these conclusions[17,40,41,42]. In the study by Hammon et al., they also identified a connection between advanced age, a raised microembolism count, and aortic sclerosis leading to “neurological deficits” as phenomena that occur after heart surgery intervention[7]. Similarly, Depenbusch writes that symptoms of acute confusion can be fostered by changes brought on by age[25]. In the keeping of a precise record of actual and potential self-care deficits, Roberts finds a strategy for avoiding these symptoms in as much as the identifying of deficits encourages thoughtfulness in nursing staff who are then expecting, and prepared to intervene, for postoperative confusion[43]. Thus, a further finding of this study is that when one is expected to reach comprehensive conclusions about acute postoperation confusion, it is vital and remains absolutely essential that various criteria such as the inclusion of measurements of both consciousness and disorientation, made on a regular basis, be a key part of the study. Patients with metabolism disorders are similarly prone to be at increased risk for the development of acute confusion.

At the beginning of the era of heart surgery, the discipline led a fringe existence at the edge of nursing and medical routine, whereas today one can speak, in terms of the quality and quantity, of ubiquitous provision dispensed through 81 heart centers in Germany[27]. As a consequence, the danger of becoming acutely postoperatively confused, in addition to suffering the basic illness, is quantitatively and, for other demographic reasons, considerably higher than it was some years ago. In 1994, Geary described a hospital confinement that had to be prolonged to up to 13 days for this very reason. Hence this phenomenon is of considerable relevance to people working in health care who are forced by hospital guidelines set up to ensure quality, and in spite of fiscal constraints, to keep hospitalization as short as possible, even for those patients who are at risk. This means that the identification of high-risk groups by means of interdisciplinary investigations into prevalence and incidence makes a contribution towards influencing the morbidity and possibly even the mortality rate of heart surgery patients in a positive direction.

## ACKNOWLEDGMENTS

The authors acknowledge the contributions, not only to this study specifically, but to nursing science in general, of the late Georges Evers, PhD, RN, Professor of Nursing Research, Catholic University of Leuven, Belgium and Universität Witten/Herdecke, Germany. The loss of such a theorist and researcher is a profound one to the health care professions.

This study was supported by a grant from the Robert Bosch Foundation, Stuttgart, Germany.

## BIOSKETCHES

**Professor Osterbrink** received his education in nursing and anesthesia at Uni-Klinik Münster in Germany. He was awarded the MN degree from the University of Glasgow, UK and the PhD from Catholic University of Leuvan, Belgium. His current research interests include pain management and he is currently Principal Investigator on the multi-center study “Pain Free Hospital” that is quantifying, for the first time, the effectiveness of pain treatment in German hospitals.

**Professor McDonough** obtained his doctoral degree with a major in psychotherapy from Drake University in Des Moines, Iowa, USA. In addition he has a MS and an MSN degree from Drake University and the University of Tennessee and a Certificate of Advanced Graduate Specialization in Family Health Nursing from the State University of New York. His current research interests include the team approach to the prevention and treatment of complications associated with the administration of anesthesia.
